# Size vs. Stereo in Illusory Depth Inversion

**DOI:** 10.3389/fpsyg.2021.817745

**Published:** 2022-02-07

**Authors:** Juno Kim, Grace Hong, Stuart Anstis

**Affiliations:** ^1^School of Optometry and Visual Science, University of New South Wales, Kensington, NSW, Australia; ^2^Department of Psychology, University of California, San Diego, La Jolla, CA, United States

**Keywords:** vision, Ames, illusion, size, depth, stereopsis, 3D shape

Judgments of object form and motion require accurate depth perception, based upon cues such as size perspective and stereo, and the more consistent these cues are the more confident are our depth judgments. However, any conflicts between these depth cues can lead to illusions of depth reversal. With near objects looking far away and far objects looking near, the perceived depth order of objects becomes ambiguous or even reversed altogether.

To examine how conflicting depth cues can reverse the perceived depth order of objects, we present novel displays showing depth inversion. We compare these to the well-known spinning ballerina (Kayahara, 2003) and the Ames window (Ames, 1951; Ittleson, 1952).

In the ambiguous spinning ballerina, the outlines of the dancer's spinning silhouette give a compelling impression of rotation, but there are no depth cues to indicate which parts are near and which parts are far away. Upon first viewing, about 50% of observers see it rotating clockwise and about 50% see it rotating counter-clockwise. Over time, the perceived direction of rotation typically reverses after a few revolutions.

The Ames window exemplifies the importance of size cues to depth. An ordinary window frame is rectangular, with two vertical sides of the same length. However, in the illusory Ames window (Ames, 1951; Ittleson, 1952), the window is distorted into a trapezoid, with one vertical side that is two or three times the length of the opposite side. When this tapered Ames window is viewed in a frontoparallel plane, it looks like a rectangular window that is slanted in depth, with the long vertical edge appearing nearer in depth than the shorter edge.

An example of the original Ames window is presented in Figure 1 below. In all our Movies, a shape rotates at 16 rev/min (3.75 s/rev) at a frame rate of 24 frames-per-second. There are 90 frames in a full 360° rotation. All the movies in this article depict continuous rotation, but sometimes these rotations are misperceived as oscillations.

**Figure 1:**
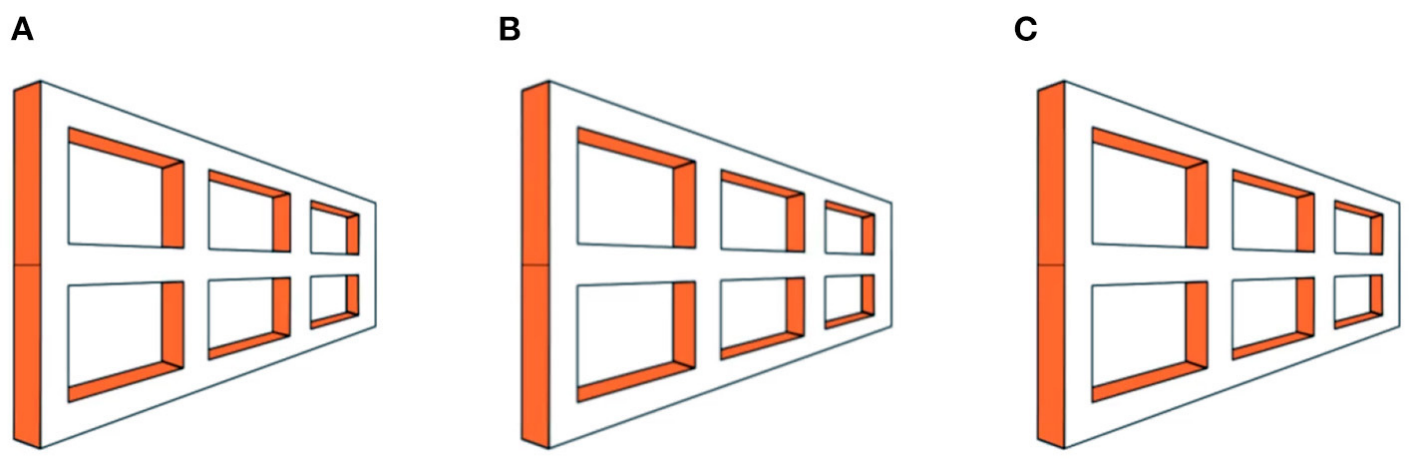
The original Ames window illusion. The window appears to oscillate back and forth over a 180° range, although it actually rotates through 360° continuously. Panels **(A)** and **(C)** are identical, while panel **(B)** contains disparity. Please see Supplementary Video 1 for an animation.

Binocular free-fusion of panels in the movies will supply a stereo signal of rotation. Each movie is a stereo pair of a rotating shape. Panels a and c are always identical, while panel b contains disparities. Thus, if a diverger views a with the left eye and b with the right eye, they will see counterclockwise rotation; whereas viewing b with the left eye and c with the right eye will yield clockwise rotation. Conversely, if a converger crosses their eyes to view b with the left eye and a with the right eye they will see clockwise rotation; whereas viewing c with the left eye and b with the right eye will yield counterclockwise rotation.

The rotating window in Figure 1 is seen veridically during the half-rotation in which the larger edge is nearer to the observer. However, during the other half-rotation, the shorter edge is nearer to the observer but still looks further away because it is optically shorter. This inverts not only the perceived depth but also the perceived direction of motion. The window appears to reverse its direction, seemingly oscillating back and forth on every half-rotation instead of rotating continuously.

Thus, size dominates over stereo in the perception of the Ames window. giving an appearance of oscillation with the longer edge always appearing nearer.

Ames (1951) attributed the illusory oscillation to our everyday experiences with “carpentered environments.” It is possible that top-down knowledge of such environments could override relative size information when inferring 3D shape and orientation. To test Ames' hypothesis, we created Figure 2 to eliminate any carpentered perspective cues. A virtual glass plate is covered with black disks that are graded in size from largest at one end to smallest at the other end. The glass plate is then rotated around a vertical axis through 360°, as with the original Ames window.

**Figure 2:**
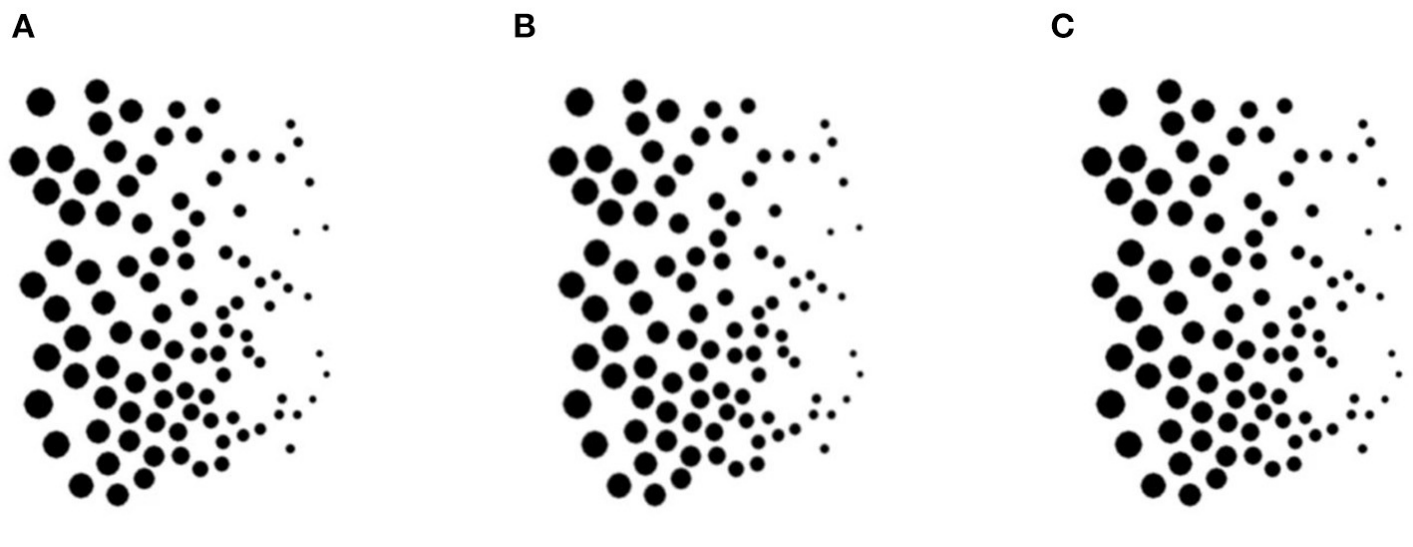
A new size gradient illusion. A stereoscopic layer of black disks appears to oscillate back and forth over a 180° range around the vertical axis, although it physically rotates 360° continuously. Size of the disks varies across the virtual glass plane. Panels **(A)** and **(C)** are identical, while panel **(B)** contains disparity. Please see Supplementary Video 2 for an animation.

The rotation is seen veridically for the half-revolution for which the large disks are closer to the observer. For the other half revolution, when the small disks are closer to the observer, they look further away. The plane of black disks seems to rotate back in the opposite direction. Like the Ames window, it appears to oscillate back and forth instead of rotating over a 360° range. Thus, relative size cues drive the experience of illusory oscillation in both demonstrations.

Ames (1951) showed that a stick thrust at right angles through the original planar Ames window will be correctly seen as rotating and paradoxically appears to “melt through” the oscillating window. We find that simulating a rod passing through our glass plane of disks achieves a similar result (see Figure 3). When the spots appear to oscillate, the rod appears to rotate continuously through 360° around a vertical axis. This effect is similar to the original Ames rod-and-window, but now there is no carpentered environment. This confirms the importance of relative size in the illusory perception of the Ames window.

**Figure 3:**
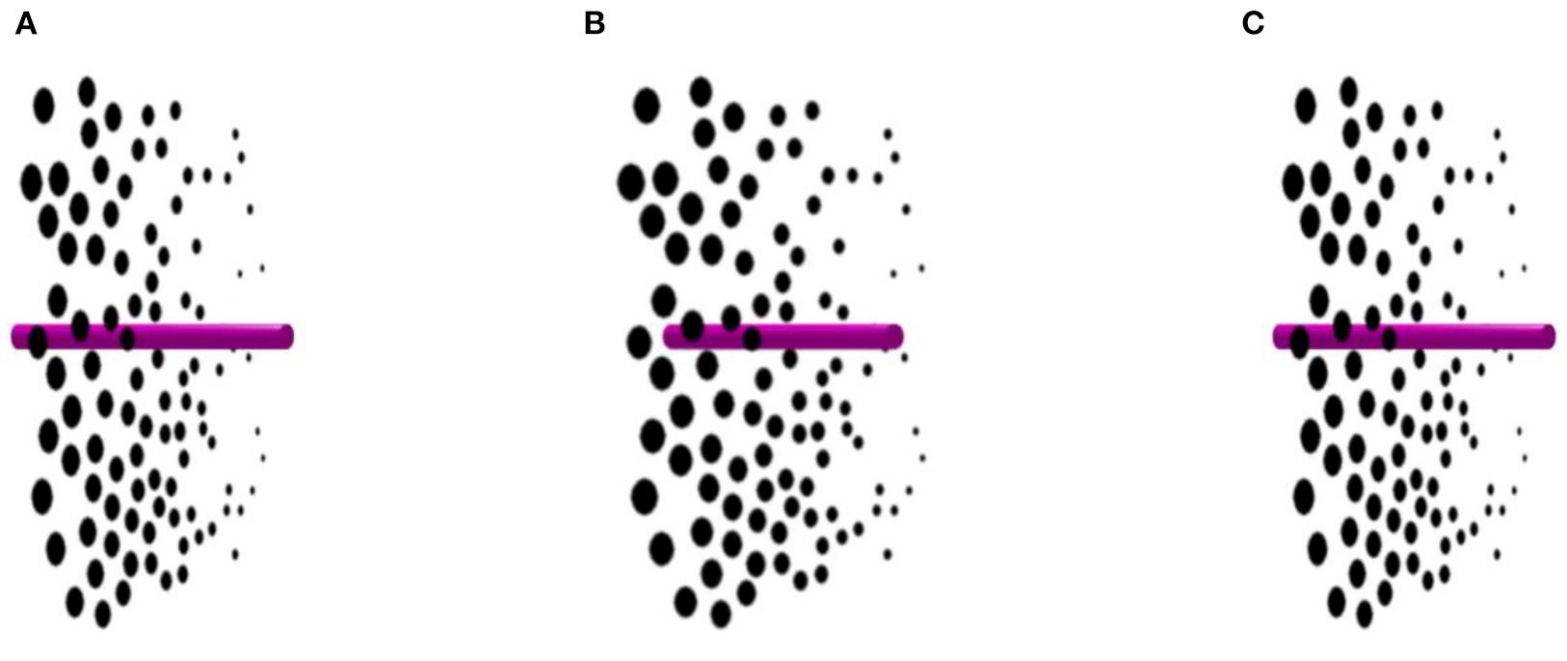
Rod through a spotted glass plane. The transparent sheet covered in dark spots appears to oscillate back and forth through 180°, but the rod appears to rotate through 360°. Panels **(A)** and **(C)** are identical, while panel **(B)** contains disparity. Please see Supplementary Video 3 for an animation.

When the stereoscopic layer of black disks oscillated continuously in Figure 2, observers perceived the spotted glass plane oscillating through an angle of only ~150° instead of a full 180°. In Figure 3, on the other hand, observers perceived the spotted glass plane as oscillating through a full 180° while the rod was rotating through a full 360°.

Why do we simultaneously perceive both a rotating rod and an oscillating spotted glass plane? It is because the window, but not the rod, contains built-in size perspective distortions that are misinterpreted as depth.

Our next animation tests whether we can re-integrate the experience of rotation in both the rod and plane as they move together. Figure 4 presents the identical motion scenario as Figure 3. However, the spots are now rendered as holes punched into a gray opaque sheet. In this scenario, the rod is only visible behind the opaque plane when it can be seen through the holes. At this point in the animation, the rod appears perceptually continuous via amodal completion, providing information about the relative depth position of both the rod and the plane. Observers viewing this animation are more likely to report that the rod and plane rotate together, and oscillation is rarely reported.

**Figure 4:**
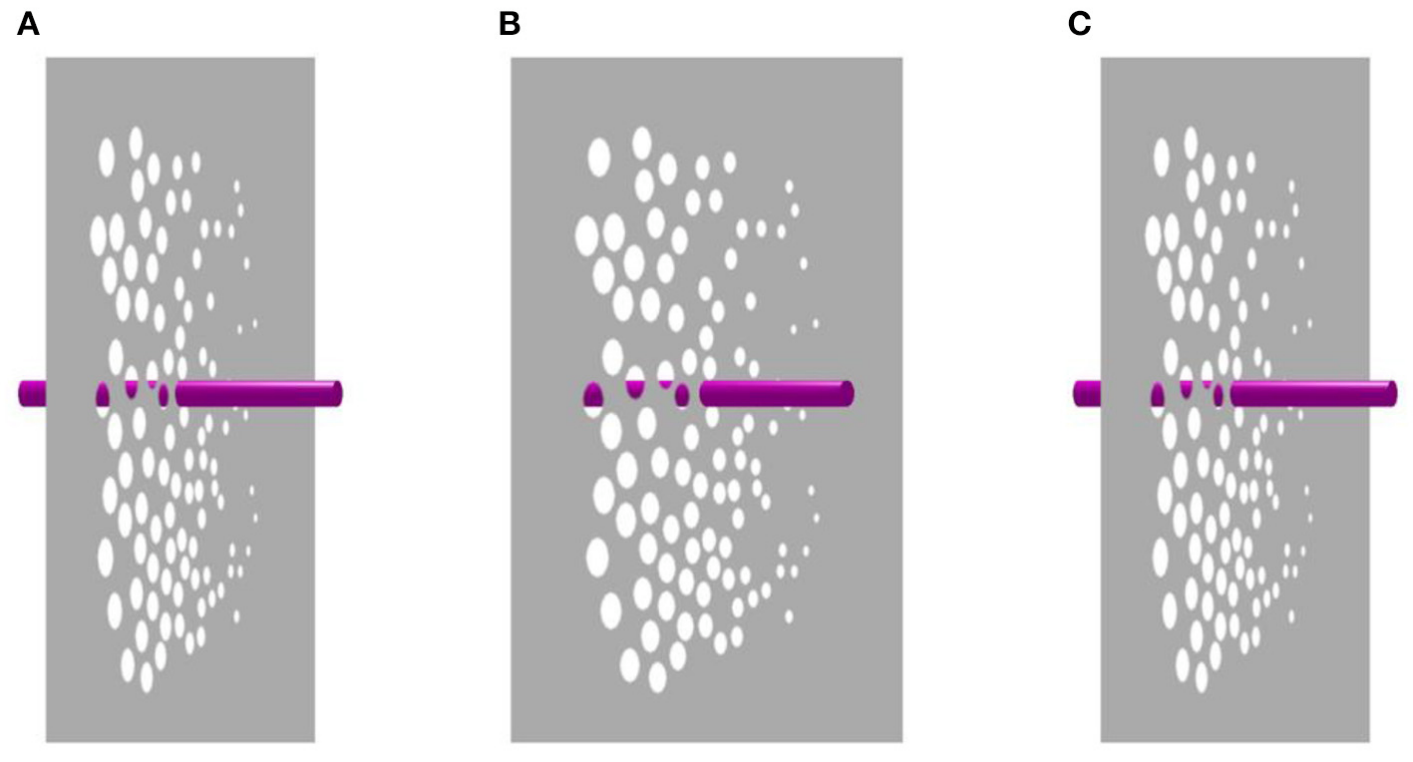
Amodal completion of the occluded rod. Both the window and the crimson rod appear to rotate through 360°. The occlusion relationships provided by the circular holes overrides relative size cues to depth. Panels **(A)** and **(C)** are identical, while panel **(B)** contains disparity. Please see Supplementary Video 4 for an animation.

Thus far, occlusion cues outweighed relative size cues in determining our judgments of depth. The absence of prominent occlusion information may explain why the rod rotates while the window oscillates in Ames' original demonstration. However, Figure 4 shows that when sufficient occlusion information is provided, the rod and the glass plane will rotate together in unison. Essentially, the rod serves as a frame of reference for the continuously rotating plane. For some observers, Figure 4 reminded them of turning a page in a book affixed to the wall.

The tapered, dotted sheet in Figures 2–4 is essentially a flat 2-D surface. For comparison, Figures 5, 6 show 3-D structures, namely wireframes in the form of truncated pyramids. These act quite differently. An Ames widow is a single trapezium with vertical edges of different sizes. These are perceived as edges of about the same length but lying at different distances. The truncated pyramids in Figures 5, 6 have four trapezoidal sides with unequal vertical edges, but these faces are interpreted as actual trapezia, not as rectangular faces that are slanted in depth. In other words, they do not look like Necker cubes under extreme perspective conditions—as they would do if the visual system treated them the same way as a single Ames window. By the same token, the two square faces of different sizes are perceived as a large and a small square, not as two squares of the same size at different distances.

**Figure 5:**
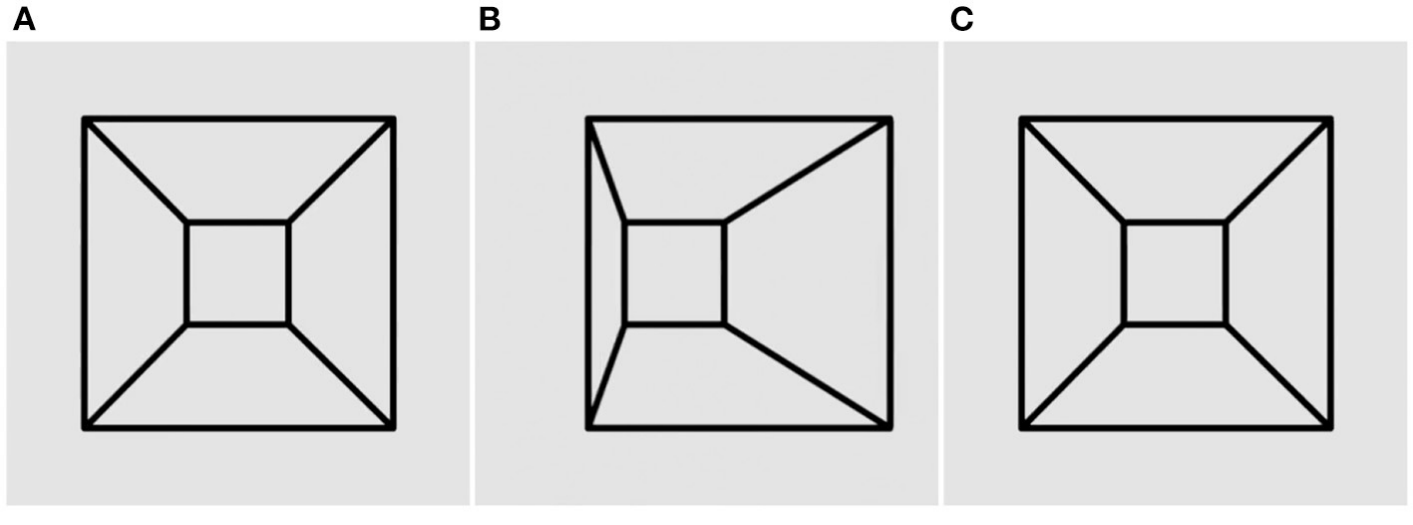
Stereo disambiguation of a trapezoidal prism. When viewed in stereo, this rotating trapezoidal prism reverses its direction from time to time, like the spinning ballerina. Sometimes the large square face looks nearer as it rotates in one direction. Sometimes the smaller face looks nearer and it seems to rotate in the opposite direction. Panels **(A)** and **(C)** are identical, while panel **(B)** contains disparity. Please see Supplementary Video 5 for an animation.

**Figure 6:**
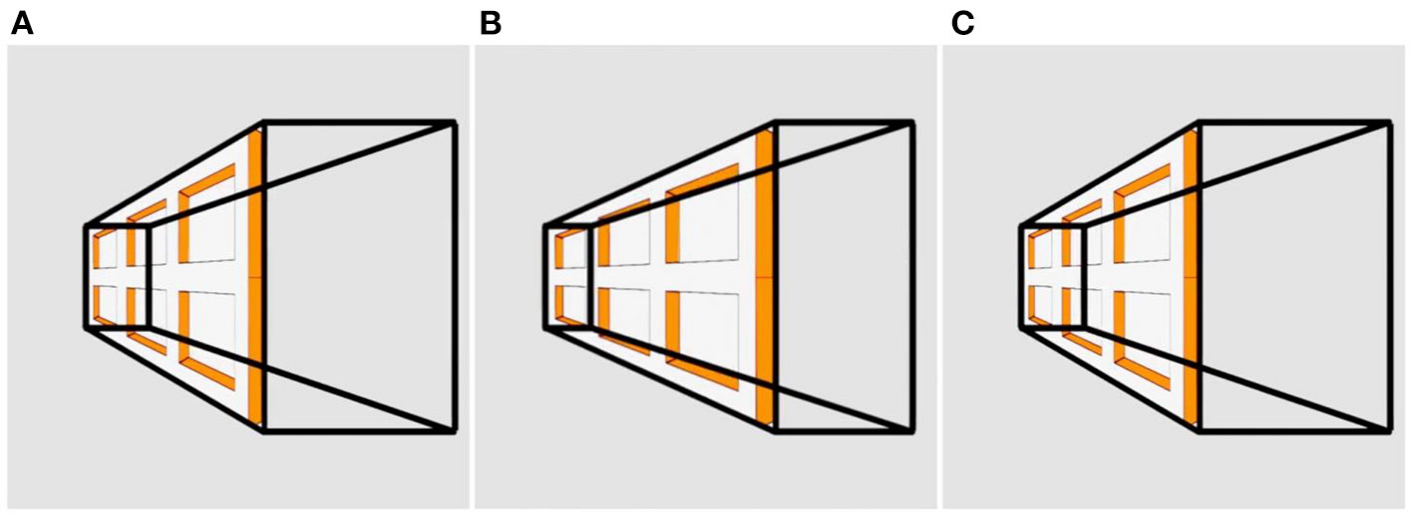
The Ames Prism. This rotating trapezoidal prism (=truncated pyramid) is textured on one side with an image of the Ames window. Panels **(A)** and **(C)** are identical, while panel **(B)** contains disparity. Please see Supplementary Video 6 for an animation.

Figure 5 shows a wire-frame trapezoidal prism (aka truncated pyramid). During both monocular and binocular viewing, the wire-frame is perceived as rotating, not oscillating. Sometimes the large square face looks nearer, consistent with the Ames window, but sometimes the small face looks nearer. The direction of perceived rotation switches from time to time, as with the spinning ballerina, depending on which face looks nearer. Unlike the Necker cube, which is inherently ambiguous, the trapezoidal prism has one of its square faces three times as large as the opposite face, and we perceive the object accurately as a truncated pyramid, and not as a rectangular prism with one of its square faces being three times as far away as the other. This size difference is interpreted as a difference in shape, not distance.

There were mixed observations toward Figure 5, which was variously seen as oscillating through 150° or as rotating through 360°. For others, the trapezoidal prism switched rapidly between oscillation and rotation. The small square might initially look farther away than the large square but then suddenly flip to looking nearer.

In Figure 6, the Ames window is texture-mapped onto one of the trapezoidal faces of the truncated pyramid. This “Ames prism” provides conflicting depth information. The wire-frame lines that construct the prism (imported from Figure 5) are informative of rotation; however, the embedded Ames window (imported from Figure 1) is informative of oscillation. The conflict between these alters the structure of the prism dynamically. During each rotation, the entire structure of the trapezoidal prism distorts continuously. It appears to oscillate non-rigidly through 45° while being turned periodically inside-out. The shape of the prism appears to change while the image of the Ames window is perceived to be moved from one side of the prism to the other, and it appears to compress and expand rhythmically like an accordion.

“Continuity of motion”—a Gestalt-like term—drives a perceptual trade-off between occlusion and transparency. At one point during rotation, the window covers parts of the wireframe and is correctly seen as an opaque surface occluding the wires. But at a later point, the wires lie in front of the window. Instead of the wires being seen to occlude the window, continuity of motion forces a perception that the wires lie behind a transparent window. Thus, occlusion is converted into transparency in the interests of perceiving smoother motion. As a result, the observer perceives the entire structure inside-out at this point.

In sum, the random-dot stimuli in Figures 2–4 show that size perspective on its own provides evidence of a swinging (oscillating) door or pane of spots without visible rotation. They also show that (*pace* Ames, 1951) neither continuous straight contours nor a carpentered environment is necessary to convert a 360° rotation into an apparent 180° oscillation. Although binocular disparity should signal an unambiguous rotation, it actually makes little difference and an illusory oscillation is still perceived.

The random-dot stimulus in Figure 2 oscillates back and forth like an Ames window, but unlike the window, it contains no angles or straight lines. The effectiveness of this random-dot demonstration rejects Ames' (1951) postulate that carpentered environments are responsible for depth inversions. Gibson (1950) noted that any size gradient in a texture served as a strong cue for seeing a depth gradient, in other words a slanted surface. We believe that an Ames window, even in a frontoparallel plane, appears to slant in depth, not because we are used to a carpentered environment but because the tapered verticals of the trapezoidal window generate an extremely clear size gradient, which in line with Gibson's prediction looks like a slanted surface. As another example, the rotating wireframe cones in Figure 7 appear to oscillate because the circular ends of the cones, simply by being larger than the cone tips, always look closer in depth to the observer. Since the two cones face in opposite directions they appear to oscillate back and forth independently as they rotate, even though they are actually rotating through 180 degrees. One can think of a cone as a distorted cylinder, in which one circular end has been shrunk into a point that defines the cone's tip. In our opinion, no prior experience with cylinders is necessary to see a depth inversion in these wireframe cones.

**Figure 7:**
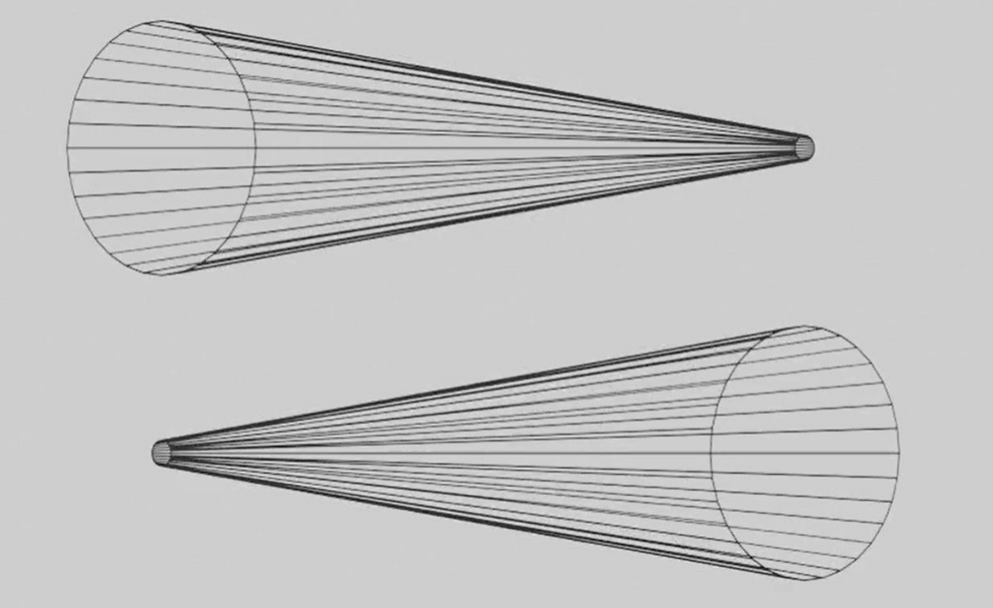
Rotating cones. These wireframe cones rotate continuously around the vertical axis, but they appear to oscillate back and forth over a 180-degree range like the Ames window. Please see Supplementary Video 7 for an animation.

In the absence of binocular cues, developmental evidence provided by Stewart (1974) indicates that infant reliance on monocular (pictorial) depth cues in the perception of the static Ames window emerges as young as 22 weeks of age. This finding was taken to support the phylogenic development of human visual sensitivity to depth cues. However, other cultural research involving the related Ames “Room” found a weaker illusion for Zambian individuals raised in remote locations mostly devoid of carpentered environments, compared with township and urban dwelling Zambian controls (Yonas et al., 1978).

These observations would suggest that exposure to carpentered environments could modulate strength of the Ames Window illusion, but our demonstrations reveal that size perspective cues are critical for the illusion to occur. All our movies looked much the same in monocular and binocular vision, so stereo made little difference in how the movies looked, and it was constantly outweighed by size perspective.

Figure 6 further highlights the complex interaction between size perspective and transparency in resolving the depth order of seen objects. In future, it would be worthwhile to further consider how the material properties of surfaces contribute to the experience of their depth order in real-world environments.

## Author Contributions

JK, SA, and GH wrote the paper and designed the video animations. All authors contributed to the article and approved the submitted version.

## Funding

JK was supported by an ARC Future Fellowship (FT140100535) and SA was supported by a grant from the UCSD Department of Psychology.

## Conflict of Interest

The authors declare that the research was conducted in the absence of any commercial or financial relationships that could be construed as a potential conflict of interest.

## Publisher's Note

All claims expressed in this article are solely those of the authors and do not necessarily represent those of their affiliated organizations, or those of the publisher, the editors and the reviewers. Any product that may be evaluated in this article, or claim that may be made by its manufacturer, is not guaranteed or endorsed by the publisher.
